# A Systems Biological Approach to Understanding the Mechanisms Underlying the Therapeutic Potential of Red Ginseng Supplements against Metabolic Diseases

**DOI:** 10.3390/molecules25081967

**Published:** 2020-04-23

**Authors:** Eunseon Jeong, Yeni Lim, Kyeong Jin Kim, Hyeon-Hui Ki, Doheon Lee, Jaehyun Suh, Seung-Ho So, Oran Kwon, Ji Yeon Kim

**Affiliations:** 1 Department of Food Science and Technology, Seoul National University of Science and Technology, Seoul 01811, Korea; todayjung@hanmail.net; 2 Department of Nutritional Science and Food Management, Ewha Womans University, Seoul 03760, Korea; ynlim@ewha.ac.kr (Y.L.); orank@ewha.ac.kr (O.K.); 3 Department of Nano Bio Engineering, Seoul National University of Science and Technology, Seoul 01811, Korea; lovelyj714@naver.com; 4 Bio-Synergy Research Center, Daejeon 34141, Korea; hhki@kaist.ac.kr (H.-H.K.); doheon@kaist.ac.kr (D.L.); 5 Department of Bio and Brain Engineering, Korea Advanced Institute of Science and Technology (KAIST), Daejeon 34141, Korea; 6 R&D Headquarter, Korea Ginseng Corporation, Daejeon 34128, Korea; seojae52@kgc.co.kr (J.S.); biopro@kgc.co.kr (S.-H.S.)

**Keywords:** metabolic disease, network analysis, *Panax ginseng*, red ginseng, systems biology

## Abstract

Red ginseng has been widely used in health-promoting supplements in Asia and is becoming increasingly popular in Western countries. However, its therapeutic mechanisms against most diseases have not been clearly elucidated. The aim of the present study was to provide the biological mechanisms of red ginseng against various metabolic diseases. We used a systems biological approach to comprehensively identify the component-target and target-pathway networks in order to explore the mechanisms underlying the therapeutic potential of red ginseng against metabolic diseases. Of the 23 components of red ginseng with target, 5 components were linked with 37 target molecules. Systematic analysis of the constructed networks revealed that these 37 targets were mainly involved in 9 signaling pathways relating to immune cell differentiation and vascular health. These results successfully explained the mechanisms underlying the efficiency of red ginseng for metabolic diseases, such as menopausal symptoms in women, blood circulation, diabetes mellitus, and hyperlipidemia.

## 1. Introduction

Metabolic diseases such as diabetes mellitus and cardiovascular disease manifest through a combination of various risk factors, such as low-grade inflammation, metabolic disorders, excess lipid accumulation, and loss of insulin sensitivity [1]. Most of all, overnutrition, lack of exercise, and obesity are major causes of metabolic disease.

Because metabolic diseases can present serious health problems, early prevention is becoming more important, and botanical products may provide health benefits [2].

*Panax ginseng* (*P. ginseng*) is commonly known as Asian or Korean ginseng. *P. ginseng* has been used in Chinese medicine as a tonic to improve stamina and vitality [3]. Previous studies revealed that *P. ginseng* has effects on cardiovascular risk factors, hypertension, type 2 diabetes mellitus, cognition, Alzheimer’s disease, and performance enhancement [4,5,6,7,8,9]. *P. ginseng* is classified into three types, depending on how it is processed: fresh ginseng, white ginseng, and red ginseng [10]. When ginseng is steamed before drying, it is called red ginseng, and its bioactivity seems to offer more potential uses than the unprocessed white ginseng roots [11]. The active constituents of red ginseng include various saponins, such as ginsenosides, a group of triterpene glycosides, polysaccharides, polyacetylenic alcohols, peptides, and fatty acid. In the steaming process, ginseng starch is gelatinized, causing an increase in saponin content. The pharmacological activity of red ginseng is attributable mainly to these ginsenosides [12,13]. The biggest difference between red ginseng and white ginseng is the presence of rare ginsenosides, which are secondary metabolites produced by the steaming process. These include ginsenoside Rh_2_, -20(S)-Rg_3_, -Rs_4_, -Rg_5_, and -Rh_4_ [14].

Clinical studies have reported that the major active components of red ginseng, such as the ginsenosides having anti-diabetes mellitus effects and anti-hypertensive effects, can improve blood flow and prevent symptoms of menopausal syndrome [15,16,17,18,19].

However, numerous disease states can only be explained by complex, multimolecular interactions rather than by the alteration of a single gene, gene product, or metabolite [20]. Thus, in order to garner a more complete and relevant understanding of the diseases, one must obtain a comprehensive perspective of the biological system, thereby uncovering the interdependent and dynamic pathways, networks, and cellular events that undergo changes as a function of disease predisposition, onset, and progression [21]. In the last 20 years, researchers have made great efforts to investigate botanical products such as red ginseng and analyze their components. Although many studies have been conducted to understand the molecular mechanisms of red ginseng, it is still unclear how the multiple components of red ginseng interact with multiple targets to exert its therapeutic effects. Investigating this effectively requires holistic analysis of the multiple components in the plant and multiple targets in humans.

In this study, we have developed a comprehensive systematic approach for understanding the mechanisms by which red ginseng acts on metabolic disease and compared the pathways involved with those affected by white ginseng using a systems biology approach [22]. This investigation enhanced our understanding of the holistic mechanisms of red ginseng.

## 2. Results and Discussion

### 2.1. Component Analysis in Red Ginseng Using Compound Combination-Oriented Natural Product Database with Unified Terminology (COCONUT) DB

The compound combination-oriented natural product database with unified terminology (COCONUT) database has been constructed to retrieve the name of herbs by scientific name. When *P. ginseng* was searched, 669 kinds of compounds were identified. Even with the same generic name, the retrieved components have a unique PubChem ID, so deduplication based on the generic name resulted in 458 components. Besides the COCONUT database analysis, there are 80 kinds of red ginseng compounds provided by the Korea Ginseng Corporation. Of these, 23 were excluded because they were not present on the COCONUT DB, and 26 were added passively. In this process, ginsenoside Rg2 of *P. ginseng* was excluded as a redundant component, and 36 kinds of red ginseng and *P. ginseng* were identified as superimposed components (Table S1).

**Figure 1:**
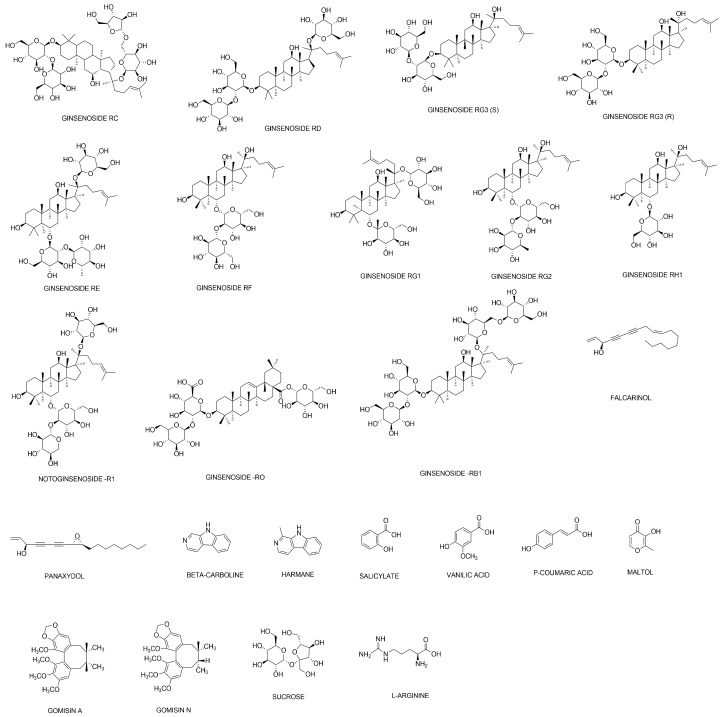
Structural formulae of components in red ginseng with their target genes.

Of the 483 components in *P. ginseng*, 168 components were identified by the COCONUT analysis to be linked with 16,569 target genes. Those compounds were regarded as the components contained in white ginseng. On the contrary, as shown in Figure 1, of the 62 components in red ginseng (which is processed *P. ginseng*), 23 components were identified that are linked with 187 target genes. Therefore, throughout this study, the networks of red ginseng and white ginseng were compared.

### 2.2. Context-Oriented Directed Associations (CODA) Network Path Exploration

There were 127,852, 98,385, 98,075, and 152,403 pathways related with menopausal symptoms in women, blood circulation, hyperlipidemia, and diabetes mellitus, respectively, that were affected by white ginseng. On the other hand, red ginseng has 3767, 2590, 210, and 1439 pathways affecting the health of menopausal women, blood circulation, hyperlipidemia, and diabetes mellitus, respectively. Among them, only the pathways related to biological processes were found because there was no complete phenotype in the phenotypes structure of context-oriented directed associations (CODA) related to menopausal symptoms and blood circulation.

To identify the most affected pathways for the same biological process, duplicate data were removed, and the biological processes were sorted by score in descending order. Many routes of analysis of the metabolic diseases affected by white ginseng identified 18 pathways associated with menopausal symptoms in women, 19 related to blood circulation, 62 related to hyperlipidemia, and 375 associated with diabetes mellitus (Table S3). On the contrary, compared to the results for white ginseng, smaller routes of analysis of the metabolic diseases of red ginseng summarized 15 pathways related to menopausal symptoms in women, 17 to blood circulation, 36 for hyperlipidemia, and 304 for diabetes mellitus (Table S4).

### 2.3. Analysis of Enrichment Pathways

For functional enrichment analysis of white ginseng, all compound-associated genes and genes of CODA network analysis routes were used as raw data. Only those that contained at least one target for a statistically significant pathway for each phenotype were selected. The gene overlaps between Pathway Studio and the CODA network were sorted in descending order, followed by *p*-value in descending order, and then duplication based on the pathway name.

For functional enrichment analysis of red ginseng, compound associated genes and genes from the CODA network analysis routes for each of the 23 components were used as raw data. A single data sheet was generated by selecting only pathways with one or more targets that were statistically significant for each phenotype. Overlapping genes between Pathway Studio and the CODA network were sorted first in descending order, then in descending order of *p*-value, and then deduplicated based on name.

**Figure 2:**
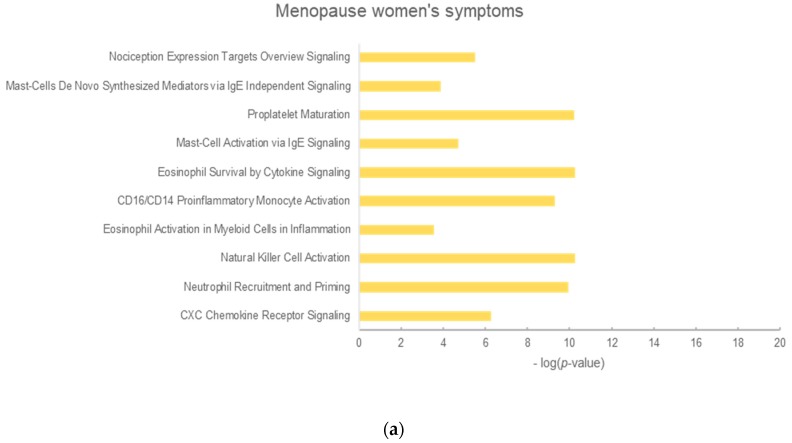

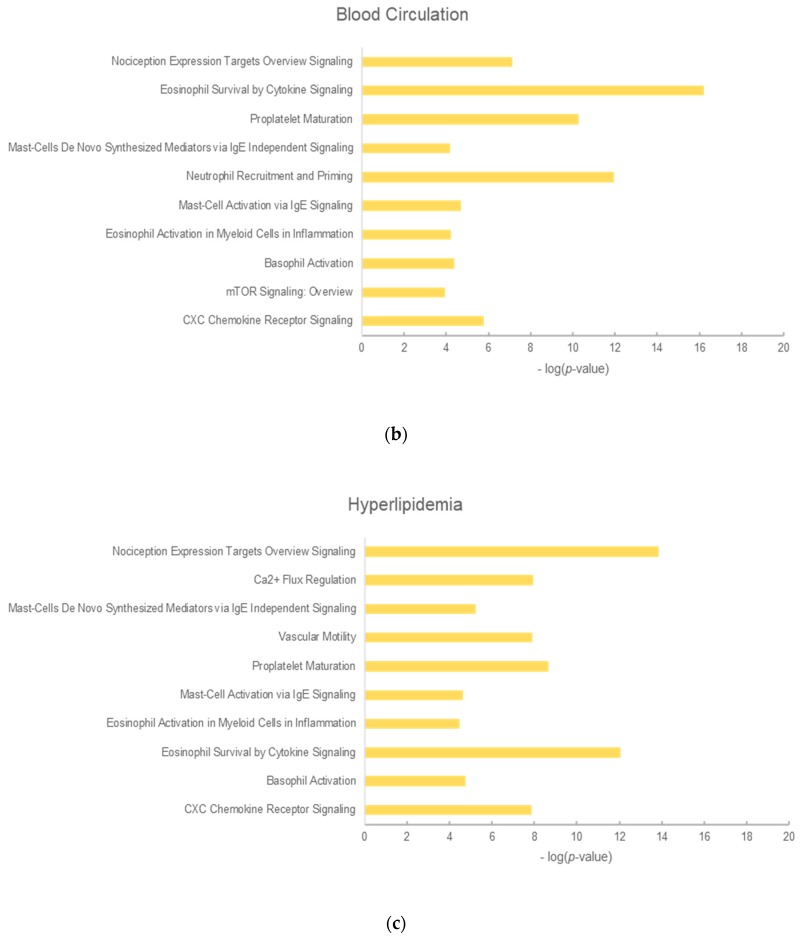

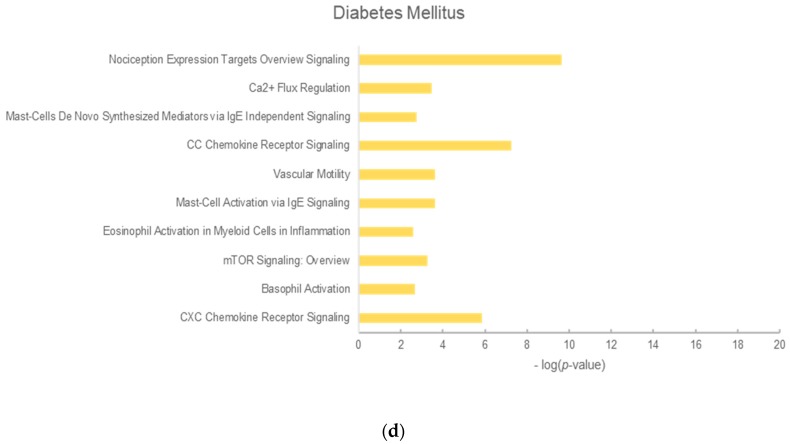
Top 10 enriched pathways of *Panax ginseng* in metabolic diseases. (**a**) Menopause symptoms (**b**) Blood circulation (**c**) Hyperlipidemia (**d**) Diabetes mellitus. The y-axis shows the significantly enriched pathways of the targets, and the x-axis shows the enrichment scores of the terms (*p* < 0.05).

**Figure 3:**
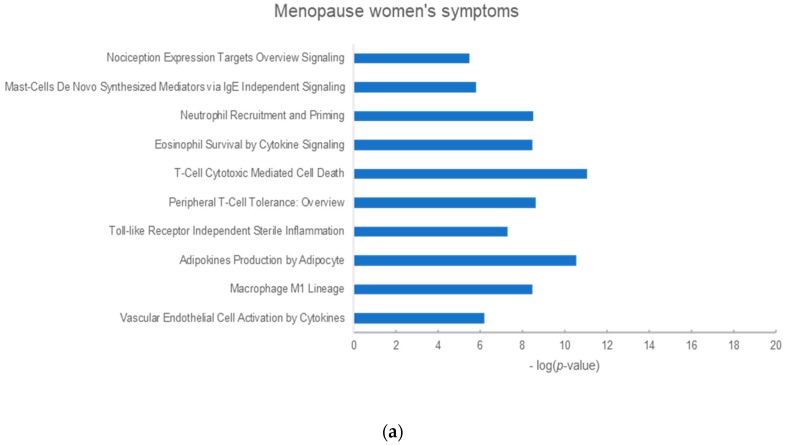

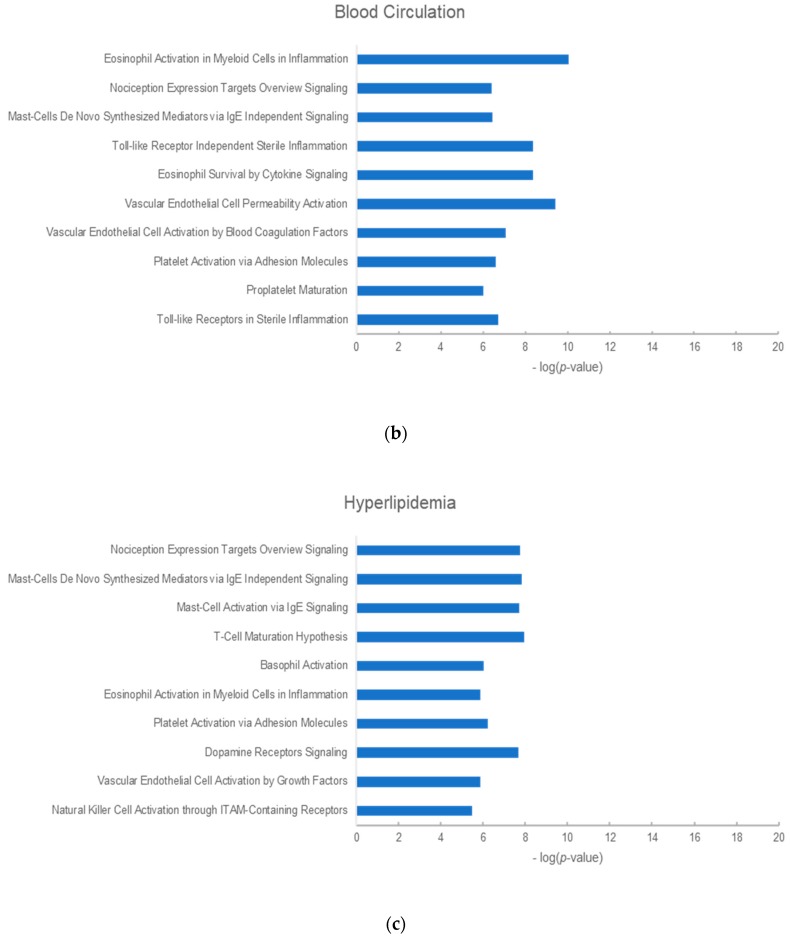

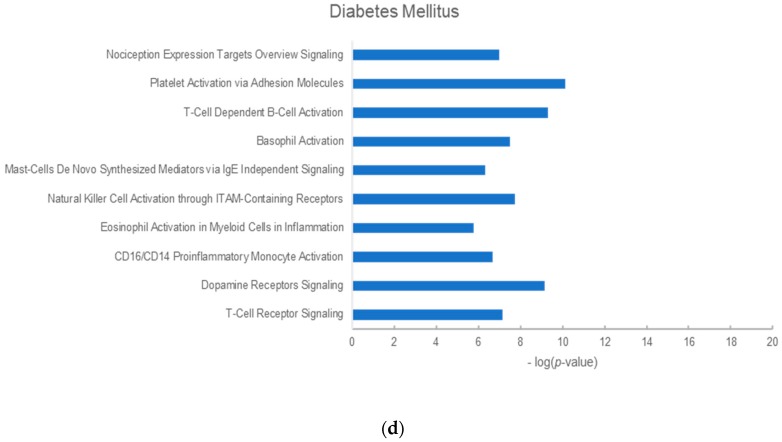
Top 10 enriched pathways of red ginseng in metabolic diseases. (**a**) Menopause symptoms. (**b**) Blood circulation. (**c**) Hyperlipidemia. (**d**) Diabetes mellitus. The y-axis shows the significantly enriched pathways of the targets and the x-axis shows the enrichment scores of the terms (*p* < 0.05).

To explore the underlying mechanisms, we selected the top 10 enrichment pathways of each phenotype related to *P. ginseng* or red ginseng (Figure 2 and Figure 3) [23].

### 2.4. Comparison of Mechanism of Action of P. ginseng and Red Ginseng

When all the pathways involved with white ginseng were combined to eliminate the duplicated pathways, a total of 101 pathways were obtained. We applied the COUNTIF function using Microsoft Office Excel 2016 to find 54 signaling pathways affected by white ginseng that are involved in all four phenotypes of interest. The Excel COUNTIF function is a statistical function that returns the number of cells within a supplied range that satisfy a given criteria. In the same way, when all the pathways involved with 23 components of red ginseng were combined to eliminate duplicated pathways, a total of 173 pathways were obtained. We applied the COUNTIF function to find 33 signaling pathways associated with red ginseng that are involved in all four phenotypes of interest.

**Figure 4:**
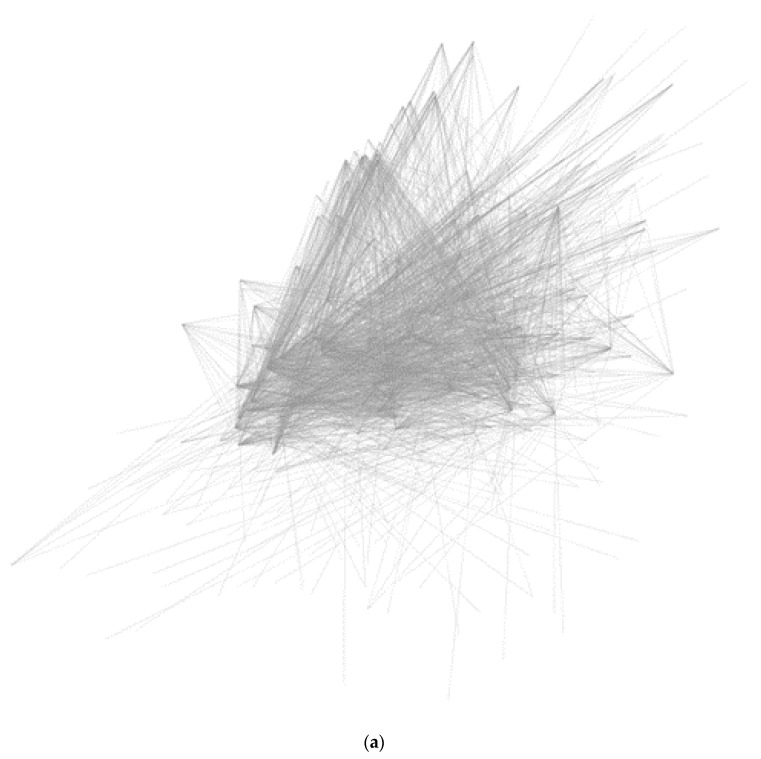

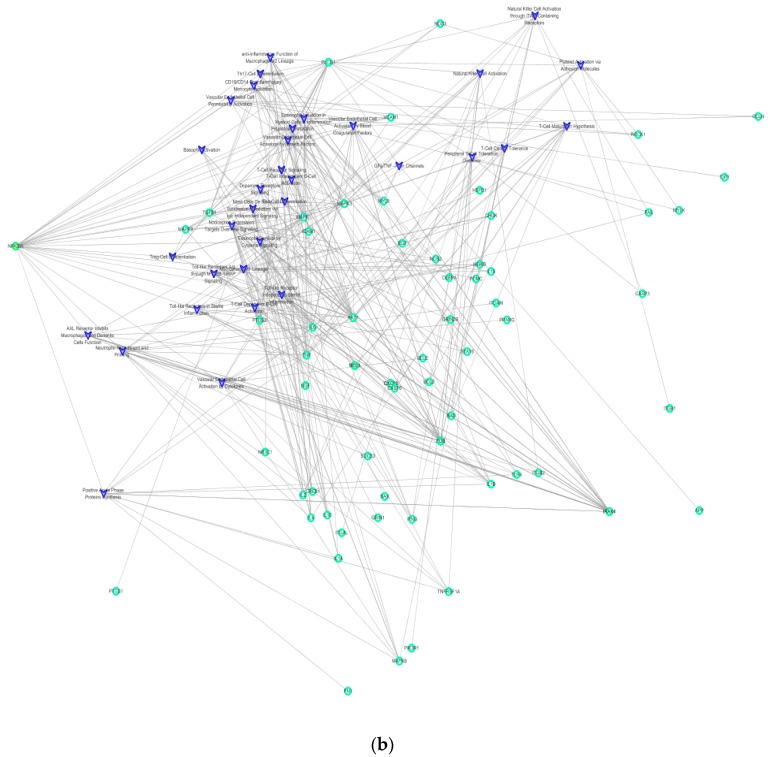
Target-pathway network. (**a**) *Panax ginseng* and (**b**) Red ginseng. Targets (green) and pathways (blue). Cytoscape file was included as supplementary information (Figures S1 and S2).

As shown in Figure 4, we constructed a T-P network involved in all four phenotypes. The network of white ginseng consisted of 806 nodes and 9396 edges, with 54 pathways and 752 targets. The red ginseng network consisted of 100 nodes and 663 edges, with 33 pathways and 66 targets. The information on white ginseng is too massive to identify the mechanisms in the network.

We identified nine signaling pathways found only in red ginseng by applying the COUNTIF function to 54 pathways of white ginseng and 33 pathways of red ginseng affecting all four phenotypes of interest (Table S5). These nine pathways mean that they are involved in one, two, or three phenotypes in white ginseng.

**Figure 5:**
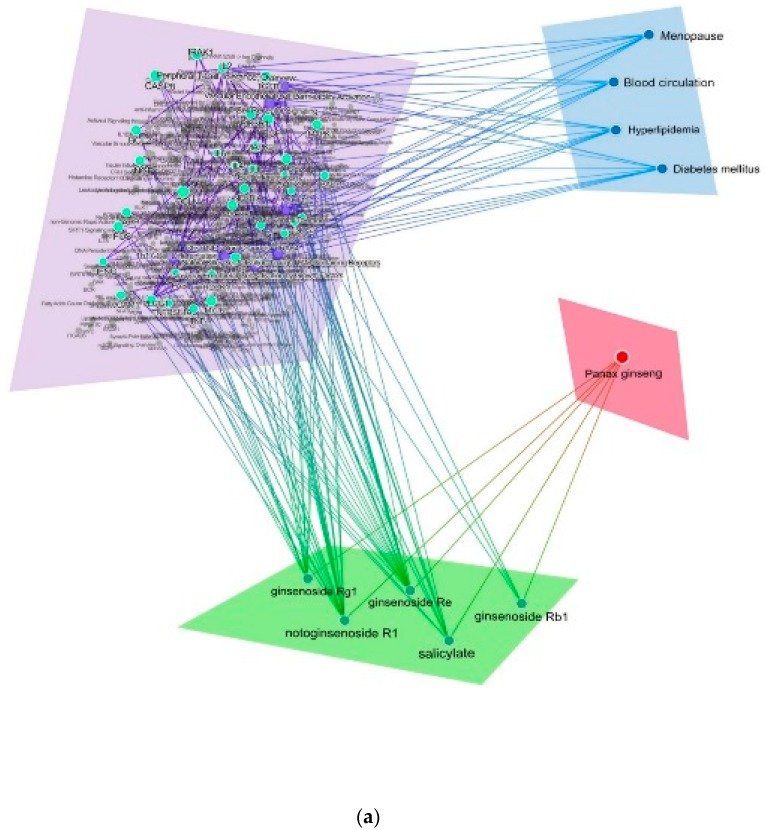

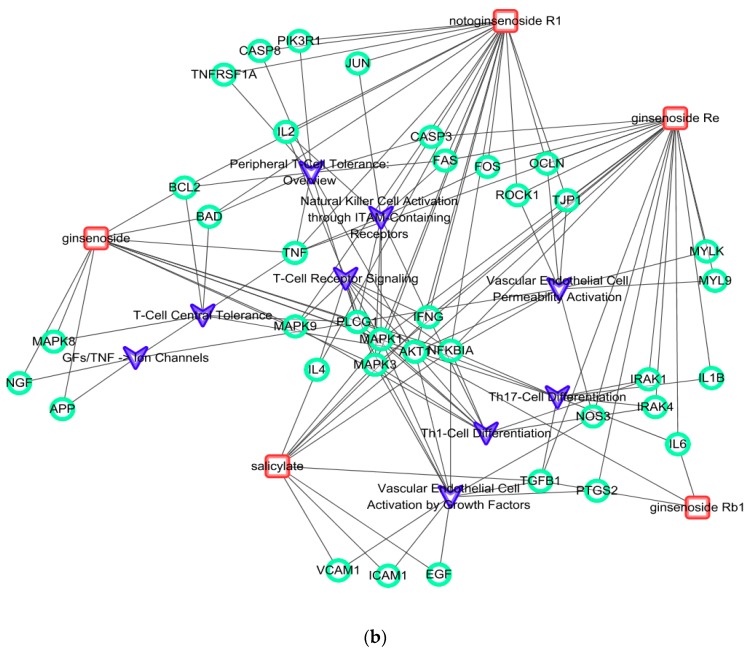
(**a**) Herb-component-target/pathway-phenotype network. (**b**) Component-target-pathway network of red ginseng. Components (red), targets (green), and pathways (blue). Cytoscape file was included as supplementary information (Figure S3).

To predict the systemic action of red ginseng, we constructed a H-C-T-P network using the CODA platform. The resulting four-layered network consisted of 459 nodes and 183 edges (Figure 5a). In white ginseng, 5099 targets, 4740 targets, 7142 targets, and 4327 targets were identified for menopausal symptoms in women, blood flow, diabetes mellitus, and hyperlipidemia, respectively (Table S6). In red ginseng, 169 targets, 142 targets, 8 targets, and 4 targets were identified for menopausal symptoms in women, blood flow, diabetes mellitus, and hyperlipidemia, respectively (Table S7).

Then, we focused and analyzed the pathways that involved only red ginseng. As a result, we identified nine pathways affected only by red ginseng with give components (ginsenoside Rg1, ginsenoside Rb1, ginsenoside Re, notoginsenoside R1, and salicylate). Among these five compounds, four compounds including ginsenoside Rg1, ginsenoside Rb1, ginsenoside Re and notoginsenoside R1 were ginsenosides. Thirty-seven targets were assigned to these nine pathways, which showed a higher degree (Figure 5b). The Vascular Endothelial Cell Activation by Growth Factors pathway exhibited the highest number of target connections (degree = 11; PLCG1, NFKBIA, MAPK3, MAPK1, VCAM1, EGF, TGFB1, AKT1, ICAM1, PTGS2, NOS3), followed by Natural Killer Cell Activation through ITAM-Containing Receptors (degree = 10; FAS, BAD, BCL2, PIK3R1, AKT1, CASP3, TNF, TNFRSF1A, CASP8, PLCG1) / Peripheral T-Cell Tolerance: Overview (degree = 10; FAS, BAD, BCL2, PIK3R1, AKT1, CASP3, TNF, TNFRSF1A, CASP8, PLCG1), T-Cell Receptor Signaling (degree = 9; MAPK9, PLCG1, NFKBIA, IFNG, IL4, IL2, MAPK3, MAPK1, AKT1) / Th1-Cell Differentiation (degree = 9; MAPK9, PLCG1, NFKBIA, IFNG, MAPK3, MAPK1, IRAK1, IRAK4, AKT1) / Vascular Endothelial Cell Permeability Activation (degree = 9; NFKBIA, NOS3, ROCK1, MYL9, TJP1, MYLK, OCLN, AKT1, PLCG1), Th17-Cell Differentiation (degree = 8; PLCG1, NFKBIA, IL1B, IL6, IRAK1, IRAK4, TGFB1, AKT1), T-Cell Central Tolerance (degree = 5; PLCG1, MAPK8, AKT1, BAD, BCL2), and GFs/TNF Ion Channels (degree = 4; PLCG1, NGF, APP, TNF). These genes related to each pathway were searched by using COCONUT DB. COCONUT DB obtained information for genes associated with compounds, which were directly connected to compounds, from the Comparative Toxicogenomics Database (http://ctdbase.org/). Although this analysis showed the overall genes and pathways related to ginsenosides, direct target protein or receptors to each ginsenoside could not be identified. Nevertheless, the network demonstrated that ginsenoside Rg1 (G-Rg1) was directly connected with AKT1, APP, BAD, BCL2, MAPK1, MAPK3, MAPK8, MAPK9, NFKBIA, NGF, PLCG1, and TNF, related to anti-apoptosis and blood glucose regulation metabolism [24,25]. Ginsenoside Rb1 (G-Rb1) was involved in inflammation through regulation of AKT1, IL6, and TNF-α [26]. Ginsenoside Re (G-Re) were linked to AKT1, CASP3, FAS, FOS, IFNG, IL1B, IL6, IRAK1, IRAK4, MAPK1, MAPK3, MYL9, MYLK, NFKBIA, NOS3, OCLN, PTGS2, ROCK1, TGFB1, TJP1, and TNF, and pathways related to anti-apoptosis and blood glucose regulation metabolism [24,25]. Notoginsenoside R1 (NG-R1) regulated AKT1, BAD, BCL2, CASP3, CASP8, FAS, FOS, IFNG, IL2, IL4, JUN, MAPK1, MAPK3, MAPK9, NFKBIA, OCLN, PIK3R1, ROCK1, TJP1, TNF, and TNFRSF1A, and these genes were related to inflammation, blood glucose regulation, and anti-apoptosis pathways [24,25,26]. Salicylate was involved in adhesion molecule production by regulating AKT1, EGF, ICAM1, IFNG, IL4, MAPK1, MAPK3, NFKBIA, TGFB1, and VCAM1 [27]. Among them, G-Re and NG-R1 were highly integrated with many different targets and signaling pathways. These results are compatible with previous studies that reported the protective effect of G-Re and NG-R1 against metabolic diseases [28,29,30,31]. Since much of the C-T interactions in the network is based on machine learning predictions without experimental validation, there is the potential for false positives on the network. To solve this drawback, we focused primarily on the top 10 targets with high orders for target analysis (Table 1). Previous studies showed that when treated with red ginseng, free fatty acid level [32], sexual function [33], menopausal symptoms, such as the Kupperman index, and lipid profiles [18] are improved in premenopausal women. Lee et al. [15] revealed that red ginseng supplementation increased levels of dehydroepiandrosterone sulfate, growth hormone, and estradiol, as well as decreased levels of glycosylated hemoglobin and insulin. It has also been reported that red ginseng intake helps blood circulation in healthy participants thorough antiplatelet action [34]. Jovanovski et al. [16] reported that red ginseng improves arterial stiffness as measured by the augmentation index. Despite studies showing no significant improvement in insulin sensitivity and postprandial plasma glucose [35,36], the antidiabetic effect of red ginseng has also been reported via improved glycemic index and insulin sensitivity [37,38,39,40,41]. The effect of reducing HbA1c and insulin resistance and the effect of reducing C-peptide and serum or whole blood glucose were also reported [42,43]. Although the functional role of red ginseng has only been demonstrated on the final phenotype, through this analysis, a list of candidates for experimental investigation for elucidating action mechanisms of red ginseng has been generated.

## 3. Materials and Methods

### 3.1. Analysis of Components in Red Ginseng

To identify the components of red ginseng and their corresponding target genes in humans, we used the COCONUT database v. 2.0 (Bio-Synergy Research Center, Daejeon, Korea) [44]. For identifying the relation between the herb and the component, components related to red ginseng were identified using the M0201 module. Besides the COCONUT database, lists of red ginseng compounds identified using in-house database (Korean Ginseng Corporation, Daejeon, Korea) were included in this analysis [22]. For identifying the relation between the herb, component, and target, the M0203 module was used.

### 3.2. Associated Phenotype Analysis

To identify the phenotypes affected by all the red ginseng target genes retrieved from the COCONUT database, we used the random walk (RW) simulator, a utility of CODA v. 2.0 (Bio-Synergy Research Center, Daejeon, Korea) [44]. The degree to which red ginseng is predicted to be affected is scored as a normalized RW score [45]:

(1) $$F_{t+1}=\alpha F_{t}A+\left(1-\alpha \right)F_{0}$$

F_0_ means the vector containing the signal amount of the start nodes. A is a matrix representing the degree of connection between each node and edge on the network. α is the rate control factor through which the signal amount is transmitted.

The starting node uses the gene associated with the red ginseng component found in the COCONUT database. The step stops when the difference between F_t+1_, F_t_, and the sum of the absolute values of the numbers of each vector becomes smaller than a certain threshold at every step (_t_). The numbers of the F_t+1_ vectors are the final RW score of the node corresponding to each.

### 3.3. Exploration of Route With Potential Targets

We searched the Path Scaffold Finder (PSF), which is a utility of CODA, to determine the route of red ginseng ingredients for efficacy against menopausal symptoms in women, blood circulation, diabetes mellitus, and hyperlipidemia. The format of each path is herb compound-compound associated gene (target)-gene target entity (biological process or phenotype).

In each path, a frequency value indicating a specific node corresponding to a gene or a biological process is assigned to each node, and a frequency value of all the nodes in the path is averaged to be scored. Compared to the total number of paths, the higher the score, the higher the frequency of the nodes containing the nodes. For each path with n nodes, the path score is calculated as follows:

(2) $$\mathrm{Path}\;\mathrm{score}=\frac{\sum_{i=1}^{n}\left(\frac{count_{paths\;with\;i-th\;node}}{count_{overall\;paths}}\right)}{count_{nodes\;in\;a\;path}}$$

### 3.4. Analysis of Enrichment Pathway

Using the results of the pathway scaffold analysis on the CODA network, we inferred the pathway that is statistically related to the four phenotypes of interest. All gene lists correspond to the compound-associated genes and genes of *P. ginseng* and red ginseng in the routes.

Functional enrichment analysis is an analytical method that identifies cell mechanisms, functions, and signaling pathways that are statistically significantly associated with a particular gene list. In this method, *P. ginseng* was regarded as an herb without distinction of components, and 23 kinds of red ginseng components, except sucrose, were deduced by each component. When uploading the gene list, the item type was set to “protein” and the type of identifiers to “name”. For each phenotype, “enriched pathway top 100” was set so that the *p*-values were sorted in descending order.

Statistical significance was analyzed by Fisher’s exact test for biological processes among the eight hit types with Pathway Studio v. 12.0.1.9 (Elservier, Amsterdam, The Netherlands).

### 3.5. Network Construction

Three networks were constructed using the CODA [44]: (1) target-pathway network (T-P network), (2) herb-component-target/pathway-phenotype network (H-C-T-P network), and (3) component-target-pathway network (C-T-P network). The overall network of red ginseng, components of red ginseng and their corresponding targets, pathway, and phenotype were employed to generate the H-C-T-P network. The pathway information of targets related to menopausal symptoms in women, blood circulation, diabetes mellitus, and hyperlipidemia were extracted from the database of Pathway Studio v. 12.0.1.9. Then, pathway enrichment analysis was performed. This four-layered network graph was constructed using the 3D modelling method of Houdini FX 16.5 (SideFX, Toronto, Canada). The C-T-P network is an extension of the C-T-P connection of the H-C-T-P Network. Signature components of red ginseng and their corresponding targets and pathways were employed to generate the C-T-P network. 

The visualized T-P/C-T-P network graphs were constructed by Cytoscape v. 3.6.1 [23].

## 4. Conclusions

To the best of our knowledge, this work is the first study to apply a systematic biology approach to investigate the molecular mechanisms of red ginseng against metabolic diseases compared with white ginseng using the CODA network. Although this study did not provide detailed mechanisms and direct target protein or receptor for each ginsenosides, this result generated an example for understanding the multiple targets of red ginseng components and their interactions. This study suggests that red ginseng extract may contribute to lowering cholesterol, controlling blood glucose, increasing blood flow, and improving the health of menopausal women through mediation of inflammation and vascular health pathways. Research is needed to verify whether the mechanisms identified in the virtual human system are exactly matched in the human body and to identify the target proteins and action pathways of red ginseng components.

## Figures and Tables

**Table 1:** Top 10 targets of red ginseng in metabolic diseases.

No.	Menopausal Women’s Symptoms	Blood Circulation	Hyperlipidemia	Diabetes Mellitus
1	TNF	MAPK1	PLCG1	PLCG1
2	AKT1	TNF	IRAK4	IRAK4
3	FOS	MAPK3	MAPK9	MAPK9
4	JUN	NFKBIA	-	-
5	MAPK1	IL6	-	-
6	MAPK3	AKT1	-	-
7	NFKBIA	FOS	-	-
8	CREB1	TGFB1	-	-
9	IL6	ROCK1	-	-
10	IRAK1	BCL2	-	-

ATT, serine/threonine-specific protein kinas; BCL2, B-cell lymphoma 2; CREB, cAMP response element-binding protein; FOS, fos proto-oncogene, AP-1 transcription factor subunit; IL, interleukin; IRAK, interleukin receptor-associated kinase; JUN, jun proto-oncogene, AP-1 transcription factor subunit; MAPK, mitogen-activated protein kinase; NFKBIA, NFKB Inhibitor Alpha; PLCG1, phospholipase C, gamma 1; ROCK1, rho associated coiled-coil containing protein kinase 1; TGFB1, transforming growth factor b 1; TNF, tumor necrosis factor alpha.

## Supplementary Materials

Table S1: List of compounds in *Panax ginseng*/red ginseng. Table S2: Associated phenotype of *Panax ginseng*/red ginseng. Table S3: Optimal routes for *Panax ginseng* to reach each biological process associated with metabolic diseases. Table S4: Optimal routes for red ginseng to reach each biological process associated with metabolic diseases. Table S5: Enriched pathways of *Panax ginseng*/red ginseng for metabolic diseases. Table S6: Gene targets of *Panax ginseng* for treating metabolic diseases. Table S7: Gene targets of red ginseng for treating metabolic diseases.
