# Supplementary figures and images for: A Systems Biological Approach to Understanding the Mechanisms Underlying the Therapeutic Potential of Red Ginseng Supplements against Metabolic Diseases
Source: Molecules. 2020 Apr 23;25(8):1967. doi: 10.3390/molecules25081967 (PMC7221703; doi:10.3390/molecules25081967)

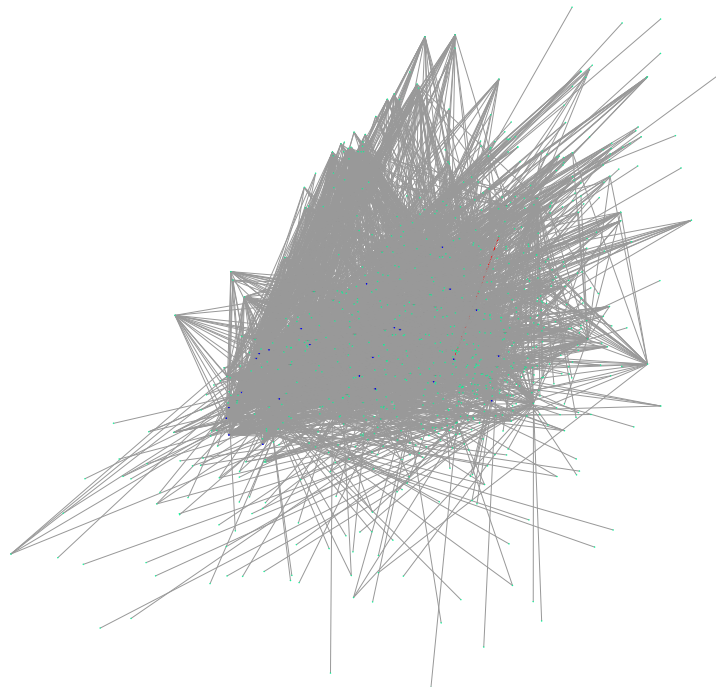

Supplement: Supplementary file 1 [file molecules-25-01967-s001.zip › Supplementary Figure 1.pdf]

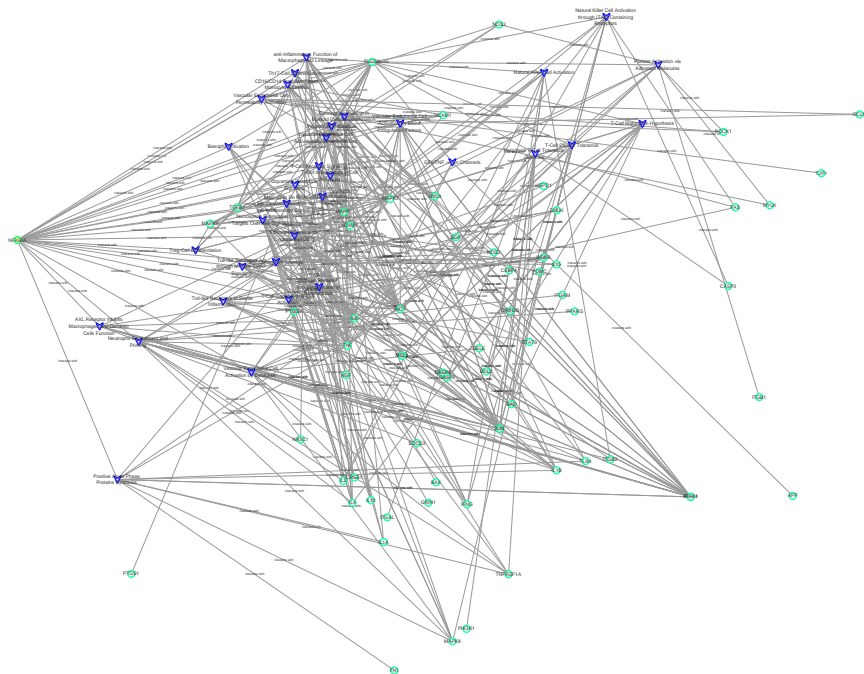

Supplement: Supplementary file 1 [file molecules-25-01967-s001.zip › Supplementary Figure 2.pdf]

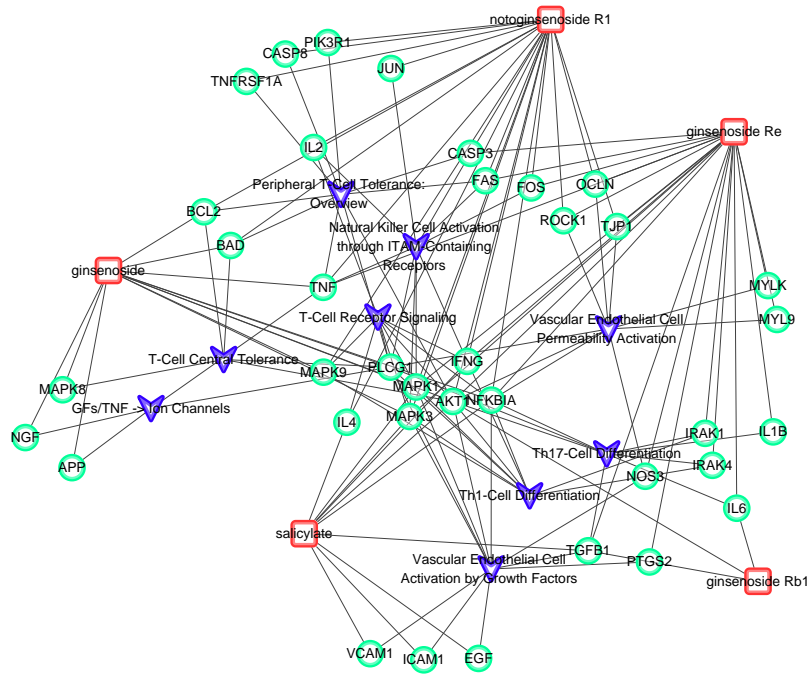

Supplement: Supplementary file 1 [file molecules-25-01967-s001.zip › Supplementary Figure 3.pdf]
